# EVALUATION OF A COMMUNITY-BASED CURRICULUM ON WELLNESS AND SAFE PAIN MANAGEMENT: A PRELIMINARY ANALYSIS

**DOI:** 10.1093/geroni/igae098.2496

**Published:** 2024-12-31

**Authors:** Gretchen Tanbonliong, Dorothea Vafiadis, Laura Plunkett, Susan Silberman, Erin Hou, Maddy Hatch, John Guidry

**Affiliations:** National Council on Aging, Arlington, Virginia, United States; National Council on Aging, Arlington, Virginia, United States; National Council on Aging, Arlington, Virginia, United States; National Council on Aging, Arlington, Virginia, United States; TRX Development Solutions, LLC, New York, New York, United States; TRX Development Solutions, LLC, New York, New York, United States; TRX Development Solutions, LLC, New York, New York, United States

## Abstract

The body of recent evidence-based interventions for pain self-management targeted at community-dwelling older adults is limited. The aim of this ongoing pilot study is to determine the efficacy of a six-week curriculum in promoting knowledge, attitudes, and behaviors related to wellness and safe pain management among older adults who were recruited via community-based organizations. Interim results from a sample survey analysis of 6 out of 8 cohorts (n=87) in a quasi-experimental crossover design showed that majority of the participants (91%) were White (63%) and Black (28%) women between the ages of 60 to 79. On a scale of 1 to 5 where “4” indicates that the knowledge or behavior is being implemented, 100% of the pain prevention strategies (i.e., maintaining a healthy weight or losing weight; engaging in physical activity; getting adequate sleep; and avoiding/limiting caffeine, tobacco, or alcohol) and two pain management strategies (“using topical therapies such as gels and creams” and “cleaning out old and expired medicines”) met this threshold post-curriculum. There were statistically significant differences observed in participants’ individual acceptance and commitment to cognitive behavior therapy (p=0.0015); adoption of meditation and/or mindfulness techniques to manage pain (p=0.0118); and reported more effective pain management (p=0.0109) between the post-curriculum and 6-week follow-up period. These preliminary findings suggest that the curriculum was an effective intervention for safe pain management among community-dwelling older adults. It has potential to be replicated across multiple community-based organizations and to target other underrepresented groups in the older adult population.

